# The Role of Power in Co‐Approaches to Health Research: Insights From Spain and the United Kingdom With a Rapid Review of Reviews

**DOI:** 10.1111/hex.70381

**Published:** 2025-10-21

**Authors:** Daniela E. Miranda, Rebecca Mead, Belén Soto‐Ponce, Magdalena Mikulak, Lois Orton, Stephanie Scott

**Affiliations:** ^1^ Department of Social Pyschology Universidad de Sevilla, Center for Community Research & Action (CESPYD) Seville Spain; ^2^ Department of Health Research Lancaster University, Faculty of Health & Medicine Lancaster UK; ^3^ Department of Sociological Studies University of Sheffield Sheffield UK; ^4^ Newcastle University, Faculty of Medical Sciences, Population Health Sciences Institute Newcastle upon Tyne UK

**Keywords:** co‐creation, coproduction, health equity, health research, power

## Abstract

**Introduction:**

Co‐approaches to health research with socially excluded groups are becoming increasingly popular in the discourse of funding schemes in Spain and the United Kingdom. Such approaches aim to challenge the traditional research paradigm between researcher–participants by sharing power in knowledge production within the parameters of academic culture. This article collates the experience of six researchers working in ongoing funded health‐related research projects that use co‐approaches alongside racialized communities, people with learning disabilities, populations involved in the criminal justice system and people experiencing deep poverty.

**Methods:**

Drawing from the authors' collective experiences and operational questions about power, a rapid review of reviews was implemented. This review included a search within five databases from April to May 2024. Findings were analysed from the Emancipatory Power Framework (Popay et al., *Health Promotion International* 36, no. 5 (2021): 1253–1263) to identify, evaluate and discover insight into power dynamics that should be understood to have meaningful impact in co‐approaches to health research, funding and evaluation of these initiatives.

**Results:**

38 articles were included in the review. A total of eight categories emerged in the analysis linked to ‘power within’, ‘power with’, ‘power to’ and ‘power over’.

**Conclusion:**

These findings contribute to deepening the critical discussion of co‐approaches, peeling back the layers of power that define academic culture, and aligning current and future health equity research with valuation of care, Open Science and new dimensions of power such as digitalization.

**Patient or Public Contribution Statement:**

This rapid review of reviews is informed by the authors' experience in co‐produced research. While patients and the public were not directly involved in conducting this review, the selection and synthesis of the literature were guided by insights from prior collaborative research with diverse communities.

## 1 Introduction

Co‐approaches have gained traction in health research funding schemes in Spain and the United Kingdom (UK), yet power‐sharing between researchers and socially excluded groups (SEG)—that is, people within a given ‘*culture, context and history at risk of being subjected to multiple discrimination’* [1]—has yet to be fully realized in academic culture [2, 3]. Co‐approaches aim to shift the research paradigm by bringing together various stakeholders to incorporate experiential knowledge in the development of relevant research for health and social policy [4], for example, coproduction, co‐creation or community‐based participatory action research (CBPAR). The values underpinning co‐approaches are deeply relational, requiring stakeholders to acknowledge and balance differing orbits of power which have their own cultures–norms, values and beliefs [5, 6]. These stakeholders can be university researchers, professionals in governmental and nongovernmental institutions (NGOs), activists in grassroots organizations and movements, and individuals living within SEG. Each manages their identities at multiple levels, navigating between historical epistemic harms and current socio‐political and economic contexts. These cultural and material dynamics influence the research process, ultimately impacting how knowledge translates into policy [7].

Using co‐approaches in academia often overestimates individual researchers' power, ignoring other structural factors and stakeholder influences that impact the research process as a whole [8]. This lack of awareness can perpetuate epistemic harms and blockade any transformational changes. Further, co‐approaches are sometimes applied tokenistically, tending to misapply or overuse related terms, that ultimately maintain researcher's control of knowledge, with SEG benefiting very little [9]. Therefore, it is necessary to scrutinize academic culture as a whole and other layers of power that shape knowledge production and contribute to sustaining epistemic harm to SEG.

This paper presents a critical rapid review of reviews, drawing on ongoing insights into the dynamics of health research within currently funded initiatives in Spain and the UK. As part of an ongoing international collaboration, the authors conduct this review to explore how power operates within co‐approaches and to situate these power dynamics within the broader ecosystem of knowledge production. The findings are intended to inform and enhance ongoing research and practice.

## 2 Centring the Role of Power in Health Research

While universities often frame themselves as neutral or progressive institutions, they actively shape the conditions under which knowledge is produced. University board meetings, internal governance structures and funding, governmental relations are ways in which political power is exerted on the research process [10]. Thus, universities should be recognized as political actors actively reproducing social dynamics through knowledge produced in relation to SEG. For example, research on Roma health in Spain and the UK must consider the historical contexts of Roma as a minority political identity. Roma became a political priority during the expansion and acquisition of eastern and central European countries [11]. Over the last two decades, initiatives linked to the EU strategic frameworks for Roma inclusion have had limited impact on Roma health and wellbeing, as policies were defined ‘for Roma, without Roma’ [12]. Similar processes affect other SEG, where research continues to perpetuate dominant narratives and values, often dictated by funding schemes that uphold historical systems. These processes reflect what Lukes describes as multi‐dimensional power: overt control through funding and agendas, covert exclusions in what is deemed valid knowledge, and deeper ideological dominance that normalizes the marginalization of SEG as knowledge producers [13]. Identifying and undoing harms requires critical engagement with the environment that governs research processes—such as who controls funding, how academic merit is defined, and whose time and knowledge are valued [14].

The dominant researcher‐participant relationship unconsciously reinforces power in meaning‐making spaces where truth and value are negotiated [15]. Researchers have the capacity to disrupt these power dynamics through the use of co‐approaches that aim to recognize the dignity, perspectives and experiential knowledge of SEG. Co‐approaches aim to uphold epistemic justice by recognizing SEG as knowers with authority in the research process [16]. A narrow view of researcher power can result in superficial responses to power‐driven barriers: simplifying language, hiring cultural liaisons or training professionals in cultural competence [17]. While these strategies may facilitate access, they rarely address structural aspects. Recent responses—such as emphasizing reflexivity and positionality—draw on feminist and critical theories [18, 19]. However, even these approaches often take a limited view without interrogating the broader cultural norms and academic hierarchies shaping research production [20]. Advancing equity demands more than the researcher's humility or goodwill in recognizing their social positions. Co‐approaches have the capacity to provide a critical response to the historical harms of knowledge production through the redistribution of epistemic authority. The following section centres on the experiences of the authors who have navigated these tensions in current research settings implementing co‐approaches.

## 3 The Researcher as an Agent of Power in Knowledge Production: Reflections From Spain and the UK

The authors have extensive experience using co‐approaches in health‐related research with various SEG, including racialized communities, people with learning disabilities, populations involved in the criminal justice system, and people experiencing deep poverty. These projects, funded by the Spanish and Andalusian Ministries of University, Research and Innovation, Wellcome Trust and NIHR School for Public Health Research, have revealed challenges at multiple levels. Authors have navigated tensions while negotiating their identities and competing demands, both within and outside academia, alongside SEG. This review emerges from shared reflexivity of these experiences and a search for strategies that are related to centring power in co‐approaches. In Table 1 the authors have collated their experiences and organized the experiences based on their own individual actions and values as researchers, interpersonal and organizational experiences with SEG partnerships, and the institutional demands of the University.

**Table 1:** Multi‐level facilitators and barriers of co‐approaches from ongoing funded projects in the UK and Spain.

	Individual level	Interpersonal‐organizational level	Institutional level
**Facilitators**	− Personally committed to justice − Sustaining long‐term relationships − Offering resources (Space, material and knowledge) − Respecting community‐held knowledge − Acknowledging historical harms − Coauthorship of papers and proposals	− Partnering with stakeholders that have differing organizational and institutional capacities (Large umbrella organizations representatives, community leaders and liaisons, activists, educators and healthcare professionals) − Acknowledging shared and competing agendas	− Increase the acknowledgement of social impact indicators in academic promotions
**Barriers**	− Emotional cost when researchers belong to the community being researched − The emotional cost of mediating between institutions and lived experiences of people in extremely vulnerable contexts	− Questioning the tokenistic nature of working with the same activists and partners that fit research culture over a long period of time − Reaching silent ranks of groups beyond organizations	− Lengthy payment processes − Low economic compensation − Bureaucratic ethical procedures − Ethical procedures dominated by research‐participant paradigms − Digitalization of protocols that create barriers for community partners − Precarious research contracts that limit capacity to influence with peers and institutions

These experiences led us to conduct a rapid review of reviews focused specifically on how power is conceptualized and addressed in the literature. To guide this analysis, we drew on the Emancipatory Power Framework [21], which articulates four interrelated dimensions of power: (1) *Power within*, the recognition of shared values and interests; (2) *Power with*, the capabilities required to build alliances and act with others to be responsive to communities' interests; (3) *Power to*, the capacities required to achieve transformative changes through decision‐making opportunities and political recognition and (4) *Power over*, the ways in which an institution holds power over a community. We used this framework to develop operational questions to guide our review and inform our ongoing research practice:

1. What roles, methods and strategies do researchers use to enhance *power within*?
2. How are community–university partnerships built and sustained to foster *power with*?
3. How can co‐approaches increase *power to* in decision‐making spaces?
4. How can *power over* be identified and challenged within co‐approaches?

## 4 Methods

The approach to this framework synthesis was a rapid review of systematic reviews, scoping reviews, meta‐analyses and narrative reviews. According to the World Health Organization, rapid reviews can support limited timelines and immediate corrective measures to project implementation while maintaining transparency [22]. Rapid reviews have been utilized as a way to quickly identify gaps and design corrective measures for ongoing initiatives. A rapid review of reviews enabled rigour, timeliness and flexibility in fast‐tracking study selection and synthesis, providing an overview of the evidence necessary for understanding the current challenges faced by the active research of the authors [23]. This allowed the authors to identify strategies and gaps across diverse studies, supporting practical, real‐time application of co‐approaches with SEG. The authors acknowledge that their background utilizing co‐approaches may have influenced the review process; however, potential biases were mitigated through the use of an analytical framework and critical, iterative processes of analysis.

### 4.1 Search Strategy

A search strategy was developed, piloted and improved with the support of a library technician from Lancaster University. The search was carried out in five databases: PsychInfo (Proquest), Web of Science, Embase, Cochrane Reviews and Scopus between April and May 2024. Two sets of descriptors were used in combination, related to co‐approaches and to health equity, as described in Table 2. Due to the initial pilot search, the authors identified the need to widen the scope of co‐approaches to include CBPAR as well as public involvement, to ensure capturing a broad range of studies that utilized co‐approaches.

**Table 2:** Search terms and combinations.

All databases		All databases
1. ‘Co’ approaches‐related terms: coproduc* OR co‐produc* cocreat* OR co‐creat* codesign* OR codesign* public involvement community participation community participatory research	AND	2. Health equity terms: health ineq* health equit* health inequal* social determinants of health

### 4.2 Study Selection and Eligibility Criteria

A Population‐Concept‐Context quality appraisal was used to define the inclusion and exclusion criteria [24]. Eligible reviews were those that centred on the *population* of Social Excluded Groups (SEG), the *concept* related to co‐approaches in research, and in the *context* of health equity and/or structural determinants of health. Table 3 outlines the inclusion and exclusion criteria of the literature found.

**Table 3:** Inclusion and exclusion criteria of the literature.

Inclusion criteria	Exclusion criteria
Focused on ‘co’ approaches with socially excluded group	Does not address ‘co’ approaches explicitly between socially excluded groups and researchers
Explore relational aspects of ‘co’ approaches in academic research	Does not explore relational aspects of ‘co’ approaches in academic research
Publications that focus on health equity and/or in addressing structural determinants of health	Does not mention any aspects of health equity and/or structural determinants of health
Publication year: after January 1, 2014	Publication year: before January 1, 2014
Language: English and Spanish	Language: any language different than English or Spanish
Accessible publication	Not accessible publication
Review publications: literature reviews, narrative reviews, scoping reviews, systematic reviews, rapid reviews, integrative reviews, meta‐analysis and meta‐synthesis	Not review and/or empirical publications: quantitative, qualitative, mixed publications, reports, book chapters, commentaries, editorials, etc.

**Figure 1:**
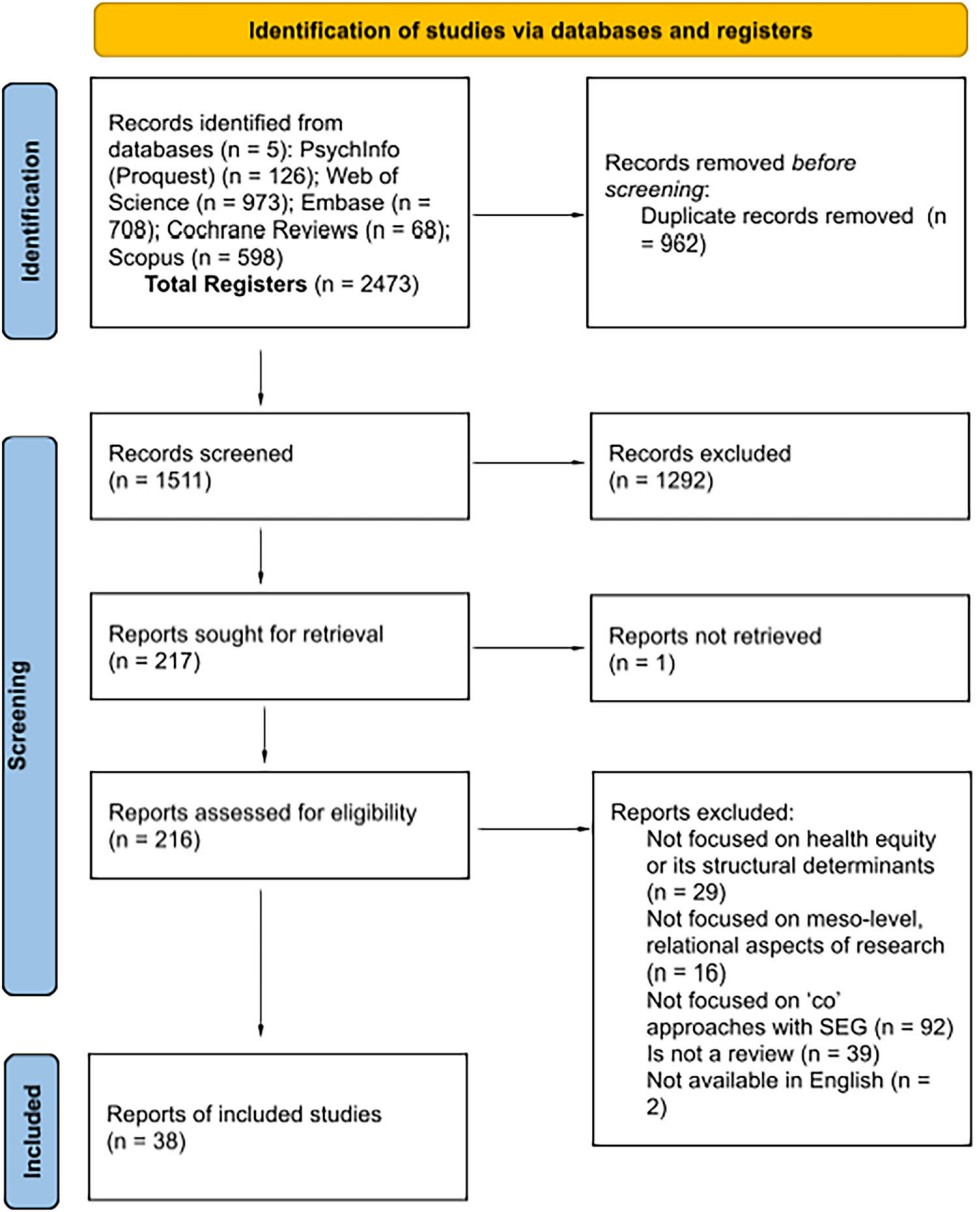
PRISMA flow diagram of rapid review screening process Page et al. [25].

The literature found was subjected to an analysis by which the bibliography that is part of this review was chosen, following PRISMA guidelines [25], see Figure 1. All database search results were included in a shared Zotero library, and a shared Excel file was utilized to track each phase of the review. Three screening processes were carried out: first, duplicated publications were excluded; secondly, a title and abstract screening was carried out by all the authors; and thirdly, a full‐text screening was implemented by five authors. Where there was uncertainty about inclusion, publications were discussed by two members of the research group, reaching an inter‐judge agreement. Moreover, each phase was reviewed by two authors to ensure consistency and reliability of findings.

### 4.3 Data Extraction and Synthesis

The analysis of the selected literature was carried out using a data chart table. Four authors were involved in this final phase through the use of a shared Excel file, which was developed and piloted by two authors involved in the data extraction phase. This chart table included the following variables: (1) authors; (2) type of publication; (3) year of publication; (4) origin of publication; (5) aim or focus of the publication; (6) researchers' roles, methods and strategies used to facilitate power‐sharing at micro level; (7) the role of university‐community partnerships when addressing power at meso level and (8) addressing hidden power at macro level. Using this type of charting table is considered a good technique to synthesize and interpret qualitative data by filtering and classifying materials according to key aspects and themes [26]. This allowed the research team to summarize findings, find common patterns and deduce themes using the Emancipatory Power Framework [21]. Data extraction and synthesis development were conducted collaboratively between two authors, with varied lived experiences in co‐approaches to research. Regular meetings were held to critically reflect on interpretations and mitigate individual bias with the rest of the authors.

## 5 Results

A total of 1511 publications were identified and screened in the search, of which 38 met the review inclusion criteria (see Figure 1 for details).

Table 4 provides the general findings with the charted information. Through a framework synthesis the authors categorized findings within the four overarching research questions. For each research question, two categories were identified, except for the question related to ‘power over’, for which three categories were identified. Below, we present the main findings.

1. **Researchers' roles, methods and strategies to facilitate ‘power within’**
  This category highlights the individual, internal factors that facilitate mutual knowledge recognition between researchers and SEG. Researchers who lead with reflexivity, positionality and care in the research process can foster more equitable, relational spaces for research.
**1.1. Practicing reflexivity and positionality.** This category focused on the processes that re‐negotiate ‘power within’ both for university and community partners. Reflexivity and cultural humility were highlighted by several authors as supporters of researchers' role capacity to develop a safe environment for co‐approaches [27, 28, 29]. Acknowledging historical factors and intergenerational traumas from colonization processes and the social environment were also identified as part of a shift in power in the research process [30, 31].
**1.2. Cultivating care in the research process.** This category centres on the emotional and relational dynamics that promote care, trust and mutual respect. Care can be understood as actions related to active listening [30], empathy and appreciation [32], a sense of generosity in collaboration [33] and cultural adaptation when appropriate [34]. Researchers' communication styles were found to be facilitators of promoting reciprocity, a sense of support and interconnectedness [28]. These were seen as fundamental aspects in the long‐term sustainability of partnerships engaging in co‐approaches [27, 35, 36].
  Our review identified what methodologies or strategies researchers could implement to facilitate fostering care and centring the knowledge community partners in co‐approaches. Among the most cited were Photovoice [30, 37, 38] and storytelling [29, 39]. Strategies based on consensus building were considered important when working to centre multiple priorities and voices between stakeholders involved [40]. Table 5 provides an overview of the methodologies and strategies researchers utilized to implement ‘co’ approaches.
  A small number of studies mentioned the use of digital tools despite reviews being conducted in the last few years [33, 51].
2. **Understanding how community–university partnerships are built and how they work to address ‘power with’**

**Table 4:** Data chart of the rapid review of reviews general findings.

Authors	Year	Focus	Power within	Power with	Power to	Power over
Acha et al.	2021	Person‐centred healthcare research with vulnerable groups	Build trust by addressing community needs; introspection and openness of the researcher; financial transparency; knowledge sharing activities; long‐term relationships	Early involvement in the process of researching and innovating; provision of feedback and recognition of the contributors' role	Action plans' strategic design and implementation; managing budgets or raising funds; making management decisions; mapping power dynamics from stakeholders	Mistrust by historical exploitation; Fear of persecution; Researchers viewed as outsiders; Historical discrimination
Brown Speights et al.	2017	Explore how CBPAR engages African American women for health equity	Researchers becoming community members and vice versa, recognizing each other's values	CBPAR provides social support and network expansion; equal investment in the research	The need to yield actionable information about what African American women need and want to promote wellness	Lack of research on the interplay between participants' social locations and its impact on research relationships, communication styles, study trajectories, knowledge outcomes, actions and framing
Coleman et al.	2023	To understand how Black and Latino sexual minority men (SMM) engage in the US‐HIV response	Researchers initiated the engagement; lack of reciprocal researcher‐community relationships	Training, healthcare resources and community programmes	Strategic planning to develop activities and structural changes	Institutional review board viewed as paternalistic; hierarchy
Cyril et al.	2015	Examine how methodological approaches maximize the effectiveness of Community Engagement	Understanding of traditional tribal and ethnic health beliefs; collaborative partnerships between community, government and academia; cultural acceptability	Building social capital; capacity building; community empowerment leading to community championship; enabling linkages with community resources; use of existing infrastructure (faith networks, tribal agency) for sustainability	Increasing the quality of local services	Tailoring community interventions versus research rigorous standardization; welfare's services insufficient capacity; hierarchy and power struggles; mistrust; inadequate programme tailoring; empowering people without infrastructural change
Epps et al.	2024	Synthesize best practices with older adults, building on three research exemplars	Be respectful; share resources; transparency and honesty; meet SEGs where they are; responsiveness; empower; recognize strengths and resources; equitable long‐term collaborations	Reliability and validity of research design and methods, accuracy and cultural humility in data interpretation; encourage communities to implement programmes; reasonable commitments	Appropriate allocation of financial resources and time should be allowed to build trusting relationships for community engagement to create the conditions where research has the potential to impact the community positively	Institutional Review Boards should also consider revising their policies to include training for community members of all ages.
Evans‐ Agnew and Rosemberg	2016	Review critically how Photovoice promotes participant voices through taking, discussing and adding text/captions	Facilitators of Photovoice	Organization of Photovoice exhibits, including the invitation to various stakeholders: policymakers, media, various public institutions	University physical spaces used as resources to display Photovoice results	Challenges with IRBs (Internal Review Boards); unclear if participants' had a choice in data analysis/selection and publication; researchers in control of Photovoice process; funding and publication restraints
Gallegos et al.	2023	Identify strategies to effectively engage, integrate and amplify potentially underrepresented populations' authentic voices in research	Critical cultural consciousness (understand culture in context of others, being ‘othered’ and ‘othering’); build trust and rapport; identify and leverage participants'/researcher' resources and skills; amplify community voices	Amplify the voices of people who are traditionally underrepresented in research and in health service delivery; equitable partnerships; social media may provide an opportunity to include people safely and discreetly in the research; informal relationship building and reciprocity; Adopt a growth mindset and be open to the reciprocal learning	Disseminate the findings and advocate accordingly	Neoliberal research paradigms prioritize researcher outputs, such as papers, funding, or individual glory, instead of social impactful research; nequitable opportunity for research participation is often underpinned by ‘structural precarity.’; cease labelling people as ‘vulnerable’
Goedhart et al.	2021	Describe and critically analyse concerns and strategies, tools and methods to support citizens living in vulnerable circumstances inclusion in health research	Mistrust; unfamiliarity with research; low confidence; unwillingness; stressors (poverty, language)	Costs and long‐time to involve hard‐to‐reach people versus usual people; discomfort with sensitive issues; differing priorities	Make space for people to talk about topics relevant to them instead of a question that matched the policy problem	Reflecting on research and policy culture in which researchers operate: how culture, structure and practices of health‐care systems need to be changed; predefined research protocols; how citizen engagement is evaluated and funded impacts researchers' latitude regarding engagement practices
Hallam‐ Bowles et al.	2022	Map coproduction approaches used in care homes for older adults to support the inclusion of residents and care staff as equal collaborators in research	Reflexivity; including all perspectives; willingness; flexibility; respecting and valuing knowledge: involvement across all stages, recognizing and using different forms of expertise; resident characteristics; limited depth of discussion; lack of self‐confidence; challenges balancing different knowledge	Reciprocity: provide support, learning, clarify expectations. Building and utilizing existing partnerships: dialogue; establish ways of working; leadership; logistical arrangements; burden and pressures on care staff; key stakeholders not involved enough	Including all perspectives and stretching new ones;	Gatekeeping; ethical procedures; delineating roles; feasibility of scaling coproduction; harms of participation
Iqbal et al.	2021	Identify and evaluate research priority setting studies with Black and Minority Ethnic groups to improve future health research	Researchers' prior consultation with community stakeholders; transparency (making transcripts available, reports, research plan, tools, key informants);	Consensus statement; research ownership	Promote community action; procedures and outcomes can inform funders and policymakers to make tailored decisions and assist researchers; quality research is required to overcome barriers to engage communities, ensure relevance of research, to address health inequalities	No plans to update research priorities; not evaluating priority setting processes; low involvement; need to incentivize stakeholders' responses; communities not informed of outcomes; tokenism
Johnson et al.	2024	Explore how research uses community‐specific physical activity measures with and for Indigenous Peoples, and how their engagement is incorporated to address health disparities	Identify Indigenous groups as communities of interest; utilize community skills and assets; cooperation; address power imbalances; sustaining relationships; commit to social change; holistic perspective of health; acknowledge community's ways of knowing, historical factors, social contexts; knowledge ownership	Providing methodological foundations to guide the implementation of culturally relevant and sustainable health‐promoting initiatives; practical approaches, to make research appropriate, respectful, and valid; involve community across all research phases	Balancing research and action for the mutual benefit of all partners; disseminating findings and knowledge to all partners; promoting long‐term process; commitment to sustainability; research impact (social change vs. academic benefits)	Not specified
Jones et al.	2018	Identify methods and approaches to engage diverse stakeholders to respond to health and environment priorities	Identify Indigenous governing organizations' roles in research; anticolonial discourse	Collaboration or partnerships with Indigenous organizations or governments	Help de‐essentializing communities and raise awareness of how colonization mediates health	Research beyond citizens being research subjects; community consent to conduct research is inconsistently identified; community permission usually inferred by researchers; Indigenous authorship is uncommon
King et al.	2022	Identify current state of the art of codesign as theory and praxis for diverse SEG	Language translation and cultural adaptation; include researchers' reflections on their learnings; collaboration with communities to ensure the intervention; digitalization of resources and platforms for engagement and adaptability	Equity was addressed including participants experiencing inequities in an area of interest, in the design of an intervention or service	Reorienting funding structures, services, access	No evaluation of desired outcomes in relation to equity; lack of consideration of power in the processes and practices of codesign (e.g. researchers' self‐critique around power)
Lebu et al.	2024	Examine existing frameworks to address negative impacts of colonialism and racism on global health, help to design global health research	Recognizing historical context; engaging local stakeholders as equal partners, intersectoral and interdisciplinary; elevating local leadership and governance; adequate time and funds; accessible meetings, times, languages; commit to redesign; streamline knowledge production, access and coauthorship; long‐term sustainability	Valuable contributions that provide insights to enhance the self‐governance and leadership capabilities of local communities, to implement programmes aimed at improving their health and general well‐being	Incorporation of a practical accountability tool to discuss the ethics of collaborative partnerships; demand accountability from funders	No diverse leadership; power imbalances in decision‐making (men vs. women; privileged vs. marginalized); not holding harm perpetrators accountable; no compensation for people's labour and time; hiring policies not inclusive; only‐online meetings or inconveniently; external‐led training; inaccessible data; no feedback; data extraction; short lead time often given in ‘requests for applications’ and ‘requests for proposals’ by donor institutions.
Lefrançois et al.	2023	Producing an inventory of existing guidelines for researchers on how to take account of sex and gender to improve health and reduce health inequities	Reflecting on one's own positionality in relation to others, who is prominent in project's results, and its impacts on actors, partners, community; use of feminist frameworks, ensures researchers', participants' and stakeholders' voices are heard	Working with a diverse partnership composition (e.g., community and governmental agencies) to ensure micro‐ and macro‐level actions; addressing partnership interactions (structure and relationships)	Monitoring knowledge mobilization; analysis of macro context, project's and partners' histories	Conventional power relations most often favour the researcher
Lokot et al.	2023	Explore literature on participatory initiatives on gender equality and Gender‐Based Violence among refugees and Internally Displaced Populations	Include local and marginalized viewpoints (vs. traditional ‘Western’ research) at each stage; encourage women and girls participation; address exploitative research; spend time with refugees and think about how best to represent their lives	Participatory approaches can increase participants' well‐being and confidence; feeling heard; opportunities for socialization; develop local strategies for change; increasing solidarity, creating transformative experiences for participants, preventing research fatigue	Women and girls less likely to participate in mixed‐gendered research; communities not used or comfortable with participating or having autonomy and voice	Power hierarchy; participation not always results in communities' meaningful participation; time and financial costs; no compensation; their knowledge contributions viewed as less valuable;
Lovo et al.	2021	Exploring how indigenous values are incorporated into Human Research Ethics, and research governance	Relationship building: respect, empathy, collaboration, sharing, reciprocity, appreciation, knowledge of culture and identity, consider time and lived experiences, humility, care and generosity, focus on empowerment, social justice, emancipatory, decolonizing, protects, gifting, knowing the language; fair open and responsible conduct; Consensus‐building with stakeholders and in accessing techniques for collaborative knowledge construction	Integrated knowledge translation activities require specific skills to engage simultaneously with the partnership process and the research process	Findings indicate the need to make recommendations for successful research which include indigenous principles and methods as part of Human Research Ethics governance; capacity building and institutional support is needed to establish Research Ethics Committees to maximize the protection of dignity and rights of indigenous peoples of Oceania	Western paradigms seen as expert knowledge versus indigenous paradigms as ‘lay knowledge’; Western ideas and methods cannot be applied understand indigenous culture; Research Ethics Committees underdeveloped or lacking; communities' lack resources; limited legal systems and expertise in bioethics
May	2024	Exploring how critical pedagogy and participatory research methods can provide useful frameworks for disabled peoples' equitable engagement and participation	Balance expert versus experiential knowledge; codesign of accessible text formats; include community as collaborators and designers; ethical underpinnings of research; democratizing design processes; equitable collaborations; self‐reflect on power imbalances; humility, faith, hope; funds of knowledge in daily routine practices and social experiences	Scholars can look towards research on inclusive education for means to challenge the ways educational institutions and structures reproduce inequality for marginalized students of various identities and experiences	Not feeling listened to or able to understand others; researchers' misunderstandings about strict hierarchies in the community, hindering participation	Research process and programme not accessible; unequal distribution of power (dominant ideology, restrictive ideas); lack of ownership of projects and findings; academics reaping material benefits
McGuffog et al.	2023	To analyse strengths and limitations researchers face when conducting Aboriginal and Torres Strait Islander health research	Supporting community‐led programmes to ensure safe research practices and cultural appropriateness; understanding local culture and contexts and being open to adapt priority research areas; providing reduced‐cost resources for services and communities	Community involvement was often recognized as an ongoing/continuous relationship, constituting a partnership rather than one off consultation	Involvement in effective knowledge translation, to inform health policy and future research that warrants appropriate funding	Attrition; inadequate implementation times; slowness of ethical approval; services' limited capacities; lack of involvement and communication; insufficient funding to consolidate relationships and disseminate results back to communities
Mehrolhasani et al.	2021	Exploring which are the challenges and interventions that promote slum residents' empowerment and health	Build community members' self‐confidence to negotiate; Build citizenship and rights awareness and sensitization; Build trust and change beliefs among residents	Civil society organizations are key actors that ensure that people living in poverty are on policy agendas; cooperation and coordination between institutions; creating and promoting organizational capacity	Provide financial assistance and training	Solutions do not address residents' needs; lack of equal opportunities to engage; low sense of responsibility; high diversity (characteristics and perceptions); negative vision of political relations; Lack of commitment, skills and interest;
Mosurska and Ford	2020	Examining how participatory approaches and community‐based research are implemented in Alaska, with special interest in the nature of community participation	Co‐analysis of information workshops	Research priorities were those previously identified in consultation and collaboration with community members/entities working on those matter; research fatigue	Reporting the research process with greater transparency demonstrates that participation is not tokenistic	Tensions between institutional values/procedures and CBPAR principles; cultural unacceptability; low adaptation to community schedules; researchers' uncomfortability to talk on behalf communities; inaccessible language; adhering to healthcare and other institutional structures can promote extractive research, foster distrust and harm, limiting researchers and communities to implement participatory; disagreements with collaborators
Ní Shé et al.	2019	Clarifying which mechanisms and resources are required to create spaces that promote the involvement of seldom heard people in health and social care research	Assure researchers' presence in community spaces to develop connections and build trust; provide financial and academic resources; share research data and outputs with community partners; ongoing education of researchers; develop flexible methods for co‐creation; budget flexibility; allocating funding to celebrate success	Processes of coproduction and codesign can promote system redesign, while overcoming prevalent challenges when engaging seldom‐heard groups in health research; facilitate ongoing feedback before, during and after the research process	Undertake an audit of involvement spaces, by all partners, before the start of the research project to ensure accessibility and continually monitor with feedback throughout the study; make university resources available to the community; share research data and outputs with community partners in an agreed and appropriate way.	Organizing activities in spaces which are not accessible for community partners; frustration when reviewing the work already done, entailing cultural inappropriate research activities; tensions between academics to adhere to project timelines and community partners to advance at their own pace; stereotypical views about researchers' motives; review ethics procedures to ensure that the competence of all partners is assumed as the default
O'Brien et al.	2020	Identifying the utility of codesign methodologies in mental health service projects with individuals and communities of culturally and linguistically diverse backgrounds	Cross‐cultural training for all researchers before engaging with communities; build equitable partnerships, adapt evidence‐based practice approaches to fit within other cultural approaches; build trust, confidentiality	Quality of relationships among researchers and culturally and linguistically diverse community affects the way communities engage in health and mental health research to overcome inequities	Rewarding co‐facilitators for their engagement	Perceived access to the projects; available communities' time, competing demands and other economic issues
Ragavan et al.	2020	Outlining how CBPR approaches can be used for developing research that addresses meaningful questions, engages culturally diverse partners and builds trust and equity with racial and ethnic minority domestic violence survivors	Creating relationships of transparency and trust; common mission; developing partnership plans; involving in community events; building on strengths, resources and interests; equal distribution of structural and individual power; training CBO staff	Equitable decision‐making and mutual accountability; confidentiality; responsiveness evolving needs and priorities of all stakeholders; shared ownership of project products	Not specified	Power inequities; language differences; absence of racial and ethnic concordance between researchers and community
Ricks et al.	2022	Identifying gaps in implementation that limit Sexual and Gender Minority community members from becoming full partners in CBPR	Academic partners have the opportunity to build community capacity by providing education and resources for navigating research processes	Low bidirectional collaboration and power sharing; community does not select research topics and/or research questions, limiting identification of priorities and outcomes important to them, promoting researcher's agenda and expertise or funder's priorities; damaging the partnership	Not specified	Limited evidence that studies prioritized removing barriers to community participation; the typical timetable for promotion and tenure at academic institutions may not be amenable to the pursuit of CBPR as this methodology is more time‐consuming than traditional research approaches
Rodriguez Espinosa and Verney	2021	Investigate CBPR as an approach for working with multicultural populations to collaboratively address relevant and impactful research questions	Value communities as key partners	Not specified	Not specified	Promotion and tenure process must value research impact including community‐engaged scholarship; lack of racial/ethnic minority researchers; CBPR not typically found in graduate level curricula; time‐intensive and requiring iterative approach; flexibility in research implementation balancing complex demands of partners and bureaucratic systems; lack of trust (institutional histories, language, communication styles); research designs unfamiliar to granting agencies; difficulty in establishing informed consent; culture of academia must encourage students and faculty to engage in CBPR; granting and accreditation agencies are increasingly demanding CBPR in both funded projects and graduate programmes; changing publication guidelines.
Rong et al.	2023	This scoping review aims to provide a guiding set of ‘best practice’ principles for community engagement in place‐based approaches	The inability to secure recurrent funding is a gap. Most articles did not detail how the community felt with the engagement or participation process. The focus of the research was on the outcomes of the project, not the engagement process itself. This is an area that researchers have indicated needs more focus; however, inflexible funding and timeline constraints make this difficult.	Researchers argue that the political and economic structures impact a person's health and wellbeing more than their individual choices; Failing to provide the longer‐term evaluation that is necessary to measure improved health outcomes and behaviour change	Short‐term funding structures; fear of being labelled an ‘informer’; burden of representing their community; stigma affecting sense of pride, sense of belonging, collective agency;	Cultural barriers; Intergenerational trauma (legacy of colonization and oppression); distrust (community members, government, researchers, stakeholders) due to past experiences; Covid‐19 messaging from their homelands, confusion, noncompliance; hierarchical and not flexible structures; tendency to engage with those who already have capacity; bias and privileged thinking
Sangill et al.	2019	Identifying how mental health service users are involved in collaborative research processes	Training and meetings to promote user researchers understanding of what was expected of them; more willing to participate in co‐designed methods; individual support and group support; learn by sharing and recognizing different experiences; negotiating precarious positions in research processes, feeling their opinions as valued by academic researchers	User‐researchers' contributions were assumed to improve all stages of the research process and were, in general, regarded as fundamental for creating new knowledge through an authentic partnership; defining different research positions; creating opportunities for decision‐making on their level of involvement; collaborative relationship	Recognition in personal, social and political aspects of their lives.	Dependency on researchers' interests; user‐researchers' unmet expectations; lack of clear expectations; lack of interest from academic researchers; less feedback than anticipated; lack of reciprocity; researchers' challenges managing their own serious mental health problems; collaborative research processes challenged by tokenism; academic language; lack of research training; academics as research authority
Schilling and Gerhardus	2017	Involving older people in health research	Provide guidance and support to people to be able to contribute meaningfully, offering training on research skills; limited confidence to contribute	Not specified	Not specified	Specific communication needs, limited mobility, temporal constraints, difficulties in bonding (lack of trust); limited continuity of participation
Siddiq et al.	2023	Community‐based mental health interventions for resettled Muslim refugees	I need for increased awareness, training and funding, to implement longitudinal interventions; changes identified at individual level and capacities to support refugees	Identified the need for researchers to collaborate with refugee communities from the inception, dissemination and sustainability of research studies and programme development	Affiliations with community advocacy and local social service agencies	No studies identified changes in broader macro‐level contexts
Smith et al.	2020	Decolonial research methodologies with Australian Indigenous Communities community members	Use Indigenous methods in data collection; engage critically in political and relational layers of reflexivity; reciprocity includes the advancement of Indigenous ways of knowing	Work in partnership with Indigenous people as key stakeholders throughout the research process that include ethical constructs of respect; reciprocity; spirit and integrity	Incorporating Indigenous perspectives in research supports political decision‐making	Data collected by colonizing methods dismisses or negates Indigenous knowledge
Soltero et al.	2021	Family‐based obesity prevention interventions among Hispanic youth	Collaborating with the community can leverage community insights and build self‐efficacy for advocacy efforts	Leveraging *promotoras* to enhance the utilization of community resources to address social determinants of health such as immigration status and direct communication with research staff	Not specified	Immigration stress and access to care or having research staff call families to discuss barriers to programme participation; language barriers; acculturation
Stotz et al.	2021	Multi‐level diabetes prevention and treatment interventions for Native people living in the United States and Canada	Including culturally relevant, culturally tailored, or culturally appropriate methods and delivery mechanisms	Consider multi‐level factors when designing interventions and evaluation strategies; community ownership of the programme has been the key to expansion of the programme; community with researchers as full and equal partners in all phases of the research process	University students involved in events; youth‐focused CBPAR interventions included policy changes.	Securing buy‐in from varying parties and competing priorities poses challenges (e.g., school vs. local grocery store priorities); social determinants specific to diabetes include access to healthful food and safe places to engage in physical activity
Riccardi et al.	2023	To measure the level of engagement reached in randomized controlled trials using community‐based participatory research in marginalized populations	Research projects should give voice to a community concern to bring academics closer to the population and enable the programme to be sustainable	The involvement of the population in data collection and data analysis is rarely used	The more a community is engaged, the more a programme can impact the social determinants of health	Participation alone is not enough because it does not erase inequities in power and resources; institutions should invest resources in partnerships as a form of trust with citizens
Thomas et al.	2023	Summarizes contemporary community‐engaged research studies incorporating a framework for improving cardiometabolic health among racial/ethnic groups	Incorporate practices that reflect community values; integration of technology	Church partnerships with academic institutions; formation of wellness community groups with peer mentoring/coaching	Material incentives as a way to ensure participation (free resources)	Substantial allocation of resources to investigators conducting co‐produced research warranted to promote sustainability; focused on micro‐level changes in behaviours and structural changes are not described
Vining and Finn	2024	Identify how photovoice has been utilized as a decolonizing methodology with indigenous communities in the United States and Canada	The review found studies where group discussions were unable to be implemented because of mobility issues, or the sensitive nature of health topics.	A consultation period with the participating community before beginning a project, and dissemination and application of findings.	Results recommend that researchers should work to develop structures that include Indigenous‐led ethics boards; partnerships should lead to follow‐up actions and policy change, although not acknowledged in publications	University ethics committees reinforce traditional research paradigms; gaps between urban and rural; lack of long‐term effects of Photovoice on addressing health equity; no studies identified structural changes
Williamson et al.	2020	Community–academic partnerships and efforts to address environmental inequities in the United States	Develop an environmental screening method that gathers data about pollution sources and maps proximity to identify the clustering of hazardous facilities	Academic‐community partnerships to jointly shape environmental justice research	One‐third of studies reported reducing the risk of exposure to environmental pollutants; a small percentage of studies encouraged enforcement of regulations	Few examples of legislative resolutions or successful prevention of industrial development
Yoeli et al.	2022	Explore how Nepal's participatory health research engages with the epistemological, methodological and ethical questions it encounters	Researchers expected to acknowledge their positionality; British team members should be competent in the Nepali language; researchers have to consider the historical validation of models in postcolonial settings through dialogical practices	Co‐approaches can increase local ownership of evidence and knowledge produced	Not specified	Dominant Western methods that centre biomedical models; research influences stigmatization of mental health; researchers are unclear on the power differentials in Nepali society; health research is largely financed by Western governments, universities and NGOs

**Table 5:** ‘Co’ method and/or strategy identified in publications.

‘Co’ Method and/or Strategy		Publications
**Facilitate dialogue and group discussion**	Consensus building methods: Delphi method, nominal group techniques	[31, 35, 40, 41]
	Community Forums	[33, 37, 38, 42]
	Critical cultural competence, aspects of accessibility (language), community‐specific activities	[29, 30, 32, 33, 36, 42, 43, 44, 45, 46, 47, 48, 49, 50]
**Qualitative research methods – CBPAR, Community Engaged Research, Citizen Science**	Focus groups	[39, 40, 42, 51]
	Interviews	[40, 50, 52]
	Storytelling, diary	[29, 30, 31, 39, 41, 51]
	Oral history	[41]
	Yarning	[29, 49]
	Lived experiences	[27, 52]
	Artistic and creative methods	[28, 39, 41, 51]
	Photovoice, Photo elicitation	[30, 31, 37, 38, 41, 53, 54]
	Body mapping	[31, 41, 50]
	Community mapping	[31, 53]
	Ethnography	[31]
	Document analysis	[41]
	Sensemaker method	[41]
	Design‐thinking	[51]
	Collaborative coding and analysis	[52, 55]
**Quantitative research methods**	Surveys	[31, 37, 40]
**Building partnerships with multi‐level stakeholders**	Community Advisory Board, coalitions	[28, 33, 42, 51, 53, 55, 56, 57, 58]
	Involving key community gatekeepers	[30, 32, 36, 37, 49, 55, 56, 57, 59, 60, 61]
	Bidirectional learning, knowledge sharing	[30, 35, 37, 38, 39, 41, 45, 59]
	Capacity‐building, workshops	[31, 32, 34, 42, 45, 47, 49, 51, 52, 53, 57, 59, 62]
	Regular meetings, ongoing interactions, communication	[27, 28, 44, 45, 49, 58]
**Involvement in research governance such as logistics and protocols**	Ethical review boards (inclusion, simplification)	[32, 37, 39, 63]
	Facilitate participants' decision‐making in all phases of research	[28, 29, 31, 32, 35, 37, 39, 40, 42, 44, 48, 52, 54, 55, 60, 61, 62, 64]
	Funding, financial compensation (including transportation costs and transparency)	[30, 35, 41, 47, 48, 59, 60]
	Adaptability, flexibility; Adjustment of timelines	[28, 30, 33, 44, 45, 47, 48, 49, 63]
	Co‐leadership in training (e.g. facilitators)	[34, 46, 47, 57, 58, 64]
	Knowledge translation, sharing and validating research results, dissemination, advocacy	[27, 28, 29, 32, 37, 42, 45, 46, 47, 51, 53, 55, 57, 61]
	Transparency, accountability	[32, 42, 47, 53, 64]

This category represents the types of partnerships that foster collective agency and embed responsiveness to community interests throughout the research cycle. The results highlight the capacity of universities, researchers and SEG communities to engage in a coordinated, mutual process.

**2.1. Building partnerships with multi‐level stakeholders.** Most studies included in the review highlight building partnerships with various stakeholders that occupy various power positions in public institutions, organizations, health and social care services, as well as various representatives from the community in the implementation of co‐approaches [33, 37, 43, 59]. A few studies suggested that collaboration can be in the form of local coalitions and research advisory boards [56]. However, how these structures are incorporated into decision‐making was not clear.

Most studies found that gaining legitimacy in the community meant involving actors that were relevant and maybe hard to reach for the researcher. For example, King et al. highlight how engaging with Indigenous youth, in partnership with Elders and adults, honoured the wisdom and leadership of the Elders in the engagement of co‐approaches [51]. Only one study discussed the selection bias in the recruitment process in co‐approaches [44]. The authors highlight how this selection bias can further hide the experience of underrepresented groups within the community itself by only accessing those who already have a certain level of education and experience with research.

A small number of studies acknowledged the role of funders as key players in co‐approaches [57]. Funders and researchers' priorities should not compete with the priorities of community partners [60]. There is a gap in discussing the explicit role of funders – both public and private – and how they influence the cohesiveness and sustainability of community–university partnerships.

**2.2. Fostering feedback and accountability across the research cycle.** The majority of studies included in this review demonstrate how co‐approaches can serve as mechanisms for mutual recognition and responsibility in research relationships across various stages of the research cycle. This includes expectations around roles and responsibilities from the outset [52], shared problem framing [55, 62], design of research protocols [33], data collection and interpretation [41, 64], feedback loops [35], evaluation [43] and dissemination [45, 46]. These practices reflect a shift away from transactional notions of research–participants, towards relationships grounded in trust, reciprocity and co‐responsibility throughout the research process.

**3. Mobilizing knowledge for ‘power to’**

This category represents the capacities required to achieve transformative changes. Results highlight the need for research to have tangible impact for SEG while the University must represent a relevant space for political engagement.

**3.1. Linking research to advocacy efforts.** Findings drawn from this rapid review highlight translating research findings into practices is essential [27, 62]. This is evident in local health policies where findings inform stakeholders [57], as well as in joint advocacy efforts with communities and other actors to drive socio‐political change [53, 61]. Nevertheless, engaging communities in advocacy actions with no perceived impacts was found as a major barrier to sustain partnerships and developed efforts [37]. The promise of material or tangible benefits through co‐approaches is questioned, suggesting a gap between research goals and actual outcomes [42].

**3.2. Transforming the university setting to a place for political engagement.** Findings describe how academic spaces can be utilized to disseminate research findings while recognizing communities' efforts. For example, Evans‐Agnew et al. suggest organizing Photovoice exhibitions in academic settings, while Lebu et al. propose to include community work within publications to be disseminated at scientific and social levels [38, 47]. This also indicates the urge to push for changes within well‐structured, rigid culture and practices of academic institutions.

**4. ‘Power over’ research governance structures.** This category examines how universities and their resources can restrict power in co‐approaches. Established hierarchies and decision‐making structures within universities often reinforce historical power imbalances, limiting the transformative capacity of co‐approaches with SEG.

**4.1. Identifying academic hierarchies.** University settings have long‐standing structures in which co‐approaches to research take place. The academic governance processes maintain paternalistic hierarchies that position public engagement in a precarious position in research [40, 56]. One study acknowledged the social value given to higher education, which in turn creates a natural hierarchy where researchers are perceived to be the ultimate authority in co‐approaches [52]. To address these imbalances, some studies suggest that providing remuneration to SEG [58], funding transportation or care [48], coauthorship [47] and expanding SEG role beyond generating data [64]. However, this overemphasis on remedial mechanisms risks reinforcing institutional control that leaves underlying hierarchies intact.

Ethical procedures were named as a limitation on the capacity of SEG in co‐approaches. Ethical protocols meant to protect participants end up reinforcing institutional authority and power imbalances. Hallam‐Bowles et al. suggest how signed consent forms reinforced unbalanced power dynamics by categorizing people as vulnerable, and deciding when and how they could participate in the research process [28]. Two reviews echoed how ethical considerations in the research process reinforced traditional research paradigms by positioning the University as the ultimate ethical authority [47, 54]. One study suggested creating an Indigenous ethical board in tandem, yet it is unclear the dynamic between the two [57].

**4.2. Understanding the researcher's influence in the university setting.** Gallegos et al. highlight that co‐approaches are not reflected in academic institutions as tensions between researcher priorities for high outputs (papers, grants) and funding does not reflect academics sensitivity to co‐approaches [33]. In fact, in some reviews, it was evident that academics struggled with adhering to rigid project timeframes were in competition with the co‐approach values of building relationships at a shared pace with multiple stakeholders [48, 49, 63]. Two studies highlighted that low investment of researcher's time in advocacy efforts were barriers to co‐approaches [50, 62]. Overall, there is a lack of critical analysis regarding researchers' recognition and influence in a larger ecosystem or how SEG perceive researchers' power within academic institutions.

**4.3. Undermining co‐approaches through historical legacies.** Researchers can still be perceived as outsiders to SEG where a history of mistrust and negative experiences between various stakeholders both within and outside the community can erode collaboration over time [50, 61, 62]. Yoeli et al. illustrate co‐approaches in the context of Nepal and the dominant biomedical model underlying Western research in the area [31]. Lebu et al. and Jones et al. suggest the need to engage in anticolonial discourse [47, 57]. There is a gap between how individual researchers engage in reflexivity and positionality (see category above related to ‘power within’) and how institutions undertake these same practices.

## 6 Discussion

This paper aimed to examine how power operates in co‐approaches to health research. First, authors collated their experiences to identify similarities across currently funded health research projects using co‐approaches with socially excluded groups (SEG) in Spain and the UK. Building on their findings, a rapid review of reviews was implemented to deepen their understanding of power dynamics and incorporate new insight into their ongoing work. The review found that academic researchers successfully working with SEG had a series of personal characteristics and values that ensured a strong relationship with multiple stakeholders. However, issues of power related to governance structures were overlooked despite being crucial for long‐term impact of co‐approaches in health research.

The review reveals how co‐approaches require care, nurturing and cultivation of real relationships over a long period of time. Most studies highlighted how researchers use personal skills and methodological strategies—such as reflexivity, positionality and inclusive knowledge practices. ‘Power within’ is built through care‐based practices that affirm the value and agency of SEG. However, there is a clear gap between the expectations placed on academic researchers by the universities—rewards of tenure, for example—and the expectations that successful co‐approaches require. This reflects a form of invisible power to shape norms, values, and what is considered legitimate knowledge or labour in academic institutions [13]. In this sense, the care work in co‐research becomes devalued because it falls outside dominant institutional logics of what counts as scientific or productive. The authors of this paper are early and mid‐career female researchers who have spent many years working alongside community partners, while some dealing with precarious academic positions in both the UK and Spain. Giddens acknowledges that agency, or the capacity to act, is dependent on access to resources and is bound by social structures such as gender, race/ethnicity and class [65]. Structural barriers such as the gendered glass ceilings in universities and underfunding of social sciences limit the transformative capacity of co‐approaches [66, 67]. Future research should examine co‐approaches' reliance on care‐work and how it impacts health equity for SEG.

The research process is an opportunity to ensure relational accountability with SEG to regain trust, transparency and relevancy of the University. Co‐approaches should move towards relational accountability grounded in mutual respect, relevance, reciprocity and responsibility [68, 69]. Public engagement in research must transcend from informing or consulting the public, towards one of citizen control and power [70, 71]. In Europe, Open Science Principles encourage citizen science and public engagement to make sure that research is responsive to societal challenges and in the hands of everyday people. Open Science moves beyond closed spaces (elite academic convenings, paywalled journals) towards invited spaces (institutionally sanctioned participatory research) [72]. The model of Open Science must go beyond occasional shared spaces between researchers and citizens to one claimed by the community. Open Science success measured through transferability capacity has been evaluated in scientists' relationship to private sector such as start‐ups and businesses as a measure of success and economic development as an indicator of understanding [73]. Future research should evaluate how Open Science principles are understood, identify practices from a power lens and encourage centring SEG as contributors and beneficiaries of innovation.

The review highlighted how the University maintains power over the researcher through limiting their capacity to influence within academic hierarchies. In this sense, Social Science relies on the parameters set by biomedical and natural sciences that shape academic governance structures that overlook the sensitive nature of relational aspects driven by co‐approaches [74]. For example, natural science represents the gold standard that dominates mechanisms of culture in academia (i.e., ethics committees and processes in acquiring adequate materials and/or compensation for partners in research). Ethical procedures were a highly cited barrier in the review. Similarly, there is a long‐standing debate opposing quantitative and qualitative research, where objectivity is tied to quantitative research and subjectivity to qualitative methods [75]. The majority of the reviews focused on qualitative approaches to research to centre SEG knowledge (see Table 5). Funding disparities further reinforce these power imbalances, restricting the allocation of resources to various types of research deemed biased, political or subjective [76]. Future research should map how power operates from resource allocation, research implementation and policy changes when implementing co‐approaches with SEG and compare with other research settings.

An important gap in the review findings was the lack of centring of digitalization in co‐approaches. Digitalization holds potential to expand participation (e.g., Power with and power to). Gallegos et al. highlighted the use of social media to engage hard‐to‐reach populations and King et al. emphasized the development of digital resources such as tools and educational resources to support co‐approaches [33, 51]. However, this gap is especially concerning given that many of the reviewed studies were conducted post‐pandemic, during a time when remote working, virtual engagement and access to digital infrastructure became central to research and service delivery. Despite significant investments in digital transitions across the public and private sectors, third sector organizations and researchers working alongside SEG must ensure that digital innovation does not reinforce existing power imbalances. Current trends often prioritize scalable, tech‐driven solutions over relational approaches—risking the reinforcement of ‘power over’ dynamics in research with SEG [77]. The review authors working in Spain have since the pandemic incorporated a digital lens into their understanding of power and access to knowledge production, since community partners had difficulty accessing digital tools [78]. This is a hidden form of power, one that maintains ‘power over’ that in some cases is yet to be explored in co‐approaches. Some research is incorporating co‐approaches into digital data infrastructures to address aspects related to ownership, control and development [79]. Future research related to co‐approaches should address digitalization as a dimension of power and its transformative capacity in health equity research.

### 6.1 Limitations

This paper has a series of limitations related to the rapid review logistics. First, the rapid review focused solely on health research, which means we missed papers that detail co‐approaches in other disciplines, which might be more developed in this area, with medical and health science being particularly rigid and resistant to change [80]. Another limitation is this was a paper about co‐approaches, but we did not include the direct involvement of the community partners in this paper. However, the experiences expressed in the background are a result of the tensions experienced from the position of researchers working in academic institutions. In fact, the process of developing this paper has been an attempt to review our own positionality as academics, engage inward as individuals and colleagues, and to name the power‐driven dynamics that we are socialized into from the beginning of our careers. This has pushed us to understand power differentials related to seniority, gender, caregiving responsibilities, race, ethnicity and national context that exist within a group of socially committed academics. The authors' next steps would be to build on findings by critically engaging outwards, incorporating the perspective of community partners and other stakeholders.

### 6.2 Conclusion

Co‐approaches in health research are in line with Open Science Principles, which aim to democratize the social power of universities and push towards a ‘responsible research and innovation’ discourse [81, 82]. These approaches to science are about both informing and ‘empowering the lay citizen to work alongside professional researchers in projects which will benefit society as a whole’ [81]. In the UK, the National Institute of Health and Care Research is increasingly expecting public engagement in all parts of the research process (see: nihr.ac.uk/pi‐standards/home). These priorities should be reflected in public and private funding schemes and how universities structure promotions. As we move the conversation forward, we suggest multi‐level changes that increase ‘power to’ of researchers in partnership with SEG in which real influence. As researchers, we must commit to collaborating with researchers from other disciplines within our universities, and beyond, to ensure shared power at the macro‐level. Partnerships across fields and shared resources will support sensitive responses to complex physical, health and social problems that are currently underway.

## Author Contributions

D.E.M. led the conceptualization, investigation, formal analysis, methodology, supervision, original draft preparation, and review and editing. R.M. contributed to conceptualization, investigation, methodology, and review and editing. B.S.‐P. contributed to methodology, investigation, formal analysis, original draft preparation, and review and editing. M.M. contributed to investigation, methodology, and review and editing. L.O. contributed to investigation and review and editing. S.S. contributed to investigation, methodology, and review and editing.

## Ethics Statement

Our study did not require ethical board approval because it did not directly involve humans or animals.

## Conflicts of Interest

The authors declare no conflicts of interest.

## Data Availability

The data that support the findings of this study are available from the corresponding author upon reasonable request.
